# The promoter of miR-663 is hypermethylated in Chinese pediatric acute myeloid leukemia (AML)

**DOI:** 10.1186/1471-2350-14-74

**Published:** 2013-07-19

**Authors:** Tao Yan-Fang, Ni Jian, Lu Jun, Wang Na, Xiao Pei-Fang, Zhao Wen-Li, Wu Dong, Pang Li, Wang Jian, Feng Xing, Pan Jian

**Affiliations:** 1 Department of Hematology and Oncology, Children’s Hospital of Soochow University, Suzhou, China; 2 Translational Research Center, Second Hospital, The Second Clinical School, Nanjing Medical University, Nanjing, China

**Keywords:** Pediatric, Acute myeloid leukemia, MiR-663, Hypermethylation

## Abstract

**Background:**

There is growing evidence supporting a role for microRNAs (miRNA) as targets in aberrant mechanisms of DNA hypermethylation. Epigenetic silencing of tumor suppressor miRNAs, including miR-663, which has recently been reported to be inactivated by hypermethylation in several cancers, may play important roles in pediatric acute myeloid leukemia (AML). However, expression of miR-663 and its promoter methylation remain status unclear in childhood leukemia.

**Methods:**

Promoter methylation status of miR-663 was investigated by methylation specific PCR (MSP) and bisulfate genomic sequencing (BGS). Transcriptional expression of miR-663 was evaluated by semi-quantitative and real-time PCR, and the relationship between expression of miR-663 and promoter methylation was confirmed using 5-aza-2’-deoxycytidine (5-Aza) demethylation reagent.

**Results:**

MiR-663 was aberrantly methylated in 45.5% (5/11) leukemia cell lines; BGS showed that the promoter was significantly methylated in three AML cell lines; methylation of miR-663 was significantly higher in Chinese pediatric AML patients [41.4% (29/70)] compared to normal bone marrow (NBM) control samples [10.0% (3/30)]. These results were confirmed by both BGS and 5-Aza demethylation analysis. In addition, miR-663 transcript expression was significantly lower in AML patients, both with and without miR-663 methylation, compared to controls; however, there were no significant differences in clinical features or French-American-British (FAB) classification between patients with and without miR-663 methylation.

**Conclusions:**

Expression of miR-663 was significantly lower in pediatric AML cells compared to NBM controls; furthermore, a high frequency of miR-663 promoter hypermethylation was observed in both AML cell lines and pediatric AML samples. Inactivation of miR-663 by promoter hypermethylation could be affected by 5-Aza demethylation. These findings suggest that hypermethylation of the miR-663 promoter may be an early event in the development of pediatric AML.

## Background

Acute myeloid leukemia (AML) is a heterogeneous clonal disorder of hematopoietic progenitor cells, which lose the ability to differentiate normally and to respond to normal regulators of proliferation [1]. Pediatric AML comprises up to 20% of all childhood leukemia. Epigenetic disturbances have been implicated in the development and pathogenesis of leukemia [2,3]. These include aberrations in methylation, which is a key epigenetic event responsible for enhanced proliferation and self-renewal, differentiation arrest, and impaired apoptosis of leukemic cells [4]. Inactivation of tumor suppressor genes by promoter hypermethylation has been increasingly recognized as a key event for leukemia, with silencing of tumor suppressor genes by aberrant DNA hypermethylation reported in hematologic malignancies, including subsets of AML [5-8]. Compared to the incidences of DNA mutations and deletions, the frequency of aberrant DNA methylation of tumor suppressor genes is high in AML. This suggests that this mechanism has a major role in the development of this rare cancer. Identifying these aberrantly methylated genes may provide better understanding of AML [9], thereby paving the way for the development of novel tumor markers and therapeutic targets.

DNA methylation consists of an enzymatic addition of a methyl group at the carbon 5 position of cytosine in the context of the sequence 5′-cytosine-guanosine (CpG), and is mediated by DNA methyltransferases (DNMT) [10-12]. The promoter regions of approximately 50% of human genes contain regions of DNA with a cytosine and guanine content greater than expected (so-called CpG islands) that, once hypermethylated, mediate gene transcriptional silencing. The following distinct roles in genomic methylation have been reported for DNMT isoforms: DNMT1 preferentially replicates already existing methylation patterns; DNMT3A and 3B are responsible for establishing de novo methylation. Abnormal expression of these methylation-related enzymes may disturb DNA methylation in pediatric AML. A common approach to study DNA methylation is to treat cells with 5-aza-2′-deoxycytidine (5-Aza) demethylation reagent. This epigenetic modifier inhibits DNA methyltransferase activity, resulting in DNA demethylation (hypomethylation), as such, treatment with 5-Aza can identify the genes that are activated by methylation.

There is growing evidence to support a role for microRNAs (miRNA) as both targets and effectors in aberrant DNA hypermethylation mechanisms. MiRNAs are noncoding RNAs between 19 and 25 nucleotides in length that regulate gene expression by inducing translational inhibition or cleavage of their target miRNAs through base pairing at partially or fully complementary sites. Several groups have shown that miRNAs are altered in human malignancies, and can function either as tumor suppressor genes or as oncogenes by regulating expression of their target genes. MiRNAs with tumor suppressor activity show similar behaviors to tumor suppressor genes, by frequently being located in deleted genomic areas or by being silenced by mutations and promoter hypermethylation in malignant cells [10,13].

MiR-663 is located at human chromosome 20q11.1 and is associated with cellular senescence, immunity, and cancer [14-18]; furthermore, multiple reports have suggested that miR-663 acts as a tumor suppressor. In human THP-1 monocytic cells and human blood monocytes, resveratrol upregulates miR-663 expression [19,20]; miR-663 decreases endogenous activator protein-1 (AP-1) activity and impairs lipopolysaccharide (LPS) induced upregulation of AP-1in THP-1 cells, in part, by directly targeting Jun B and Jun D transcripts [19]; miR-663 is further involved in a resveratrol-related pathway through targeting transforming growth factor-β1 [21]. Downregulation of miR-663 in tumor cells may contribute to aberrant cell hyperplasia, leading to the development of gastric cancer [15,22], and has been reported to induce mitotic catastrophe and growth arrest in human gastric cancer cells [15]. MiR-663 is sensitive to oscillatory shear (OS) and plays a key role in OS-induced inflammatory responses by mediating the expression of inflammatory genes in human umbilical vein endothelial cells (HUVECs) [23]. Mechanistically, miR-663 has been found to promote the cellular G1/S transition by directly targeting p21WAF1/CIP1; however, the inhibitory effects of miR-663 on the G1/S transition could be rescued by p21WAF1/CIP1 silencing [24]. In addition, miR-663 plays an important role in ATRA (all-trans retinoic acid)-induced HL-60 cell differentiation. These evidences suggest that lentiviral-mediated delivery of miR-663 could potentially be used directly as an anticancer treatment in hematological malignancies [18].

To date, there have been few reports in relation to the expression of miR-663 and the methylation status of its promoter in pediatric leukemia. In this study, we have provided the first evidence of miR-663 methylation in both AML cell lines and pediatric samples. These suggest that miR-663 may function as a tumor suppressor in pediatric AML.

## Methods

### Cell lines

Leukemia cell lines HL-60, MV4-11, U937, DAMI and K562 were obtained from the American Type Culture Collection (ATCC). CCRF, Raji , Jurkat , 697 and SHI-1 cell lines (gifts from Professor Wang Jian-Rong, The Cyrus Tang Hematology center of Soochow University). The entire cell lines were maintained at 37°C in the RPMI 1640 (Gibco^R^, Life Technologies, Carlsbad, CA) supplemented with 10% fetal bovine serum (Invitrogen, Life Technologies, Carlsbad, CA).

### Patients and samples

Bone marrow specimens were obtained at the time of diagnosis during routine clinical assessment of 70 pediatric patients with AML, who presented at the Department of Hematology and Oncology, Children’s Hospital of Soochow University between 2000 and 2010. Ethical approval was provided by the Children’s Hospital of Soochow University Ethics Committee (No. SUEC2000-021), and informed consent was obtained from the parents or guardians. AML diagnosis was made in accordance with the revised French–American–British (FAB) classification. Cytogenetic data was available in 64 patients. The main clinical and laboratory features of the patient cohort are summarized in Table 1. Additionally, bone marrow samples from 12 healthy donors and 18 patients with Idiopathic thrombocytopenic purpura (ITP) were analyzed as controls. Bone marrow mononuclear cells (BMNCs) were isolated using Ficoll solution within 2 h after bone marrow samples harvested and immediately subjected for the extraction of total RNA and genomic DNA.

**Table 1:** Correlation of miR-663 methylation with clinical features in pediatric AML patients

**Patient’s parameter**	**Status of miR-663 methylation**			
	**Methylated (n = 29)**	**Unmethylated (n = 41)**	**Total**	**P value**
**Age(median and range, year)**	6.12(1–13)	6.72(1–11)	6.47(1–13)	0.879
**Gender (male and female)**	16/13	18/23	34/36	0.602
**Laboratory parameters (median and range )**				
**WBC (10**^**9**^**/L)**	15.3(0.8-51.1)	17.2(0.8-43.6)	16.4(0.8-51.1)	0.800
**Hemoglobin (g/L)**	77.3(32–176)	70.3(32–107)	73.2(32–176)	0.203
**Platelet count (10**^**9**^**/L)**	54.2(12–310)	73.6(23–273)	65.54(12–310)	0.194
**FAB subtype, n**				
**M1**	0	12	12	
**M2**	18	14	32	
**M3**	0	10	10	
**M4**	5	0	5	
**M5**	6	5	11	0.264
**miR-663 transcript**	2.84	2.64	2.67	0.827

*Abbreviations*: *FAB:* French-American-British, *WBC:* white blood cells.

### Quantitative reverse-transcription PCR for miR-663

Quantitative real-time PCR was performed to determine the expression levels of miR-663 genes. Total RNA was reverse transcribed using the Reverse Transcription Kit, according to the manufacturer’s protocol (Applied Biosystems Inc., Foster City, CA). The reverse primers were: U6 5′-CGCTTCACGAATTTGCGTGTCAT-3′ and miR-663 5′-GTCGTATCCAGTGCGTGTCGTGGAGTCGGCAATTGCACTGGATACGACGCGGTCC-3′. The real time PCR primers used to quantify U6 expression were: F: 5′-GCTTCGGCAGCACATATACTAAAAT-3′ and R: 5′-CGCTTCACGAATTTGCGTGTCAT-3′ and for miR-663 were: F: 5′-GTGCGTGTCGTGGAGTCG-3′ and R: 5′-TTTAGGCGGGGCG-3′. Expression of miR-663 was normalized to endogenous U6 expression using the SDS relative quantification software (Applied Biosystems Inc., Foster City, CA).

### Sodium bisulphite modification of genomic DNA

High-molecular-weight genomic DNA was extracted from cell lines and biopsies by a conventional phenol/chloroform method. The sodium bisulphite modification procedure was according to the manufacturer’s instructions of EZ DNA methylation Gold Kit (http://www.zymoresearch.com, Zymo Research Corporation, Irvine, CA). Briefly 2 μg of extracted DNA was bisulphite-modified with the EZ DNA methylation Kit which converted all unmethylated cytosines to uracils and leaving methylcytosines unaltered. Modified DNA was resuspended in TE buffer (10 mM Tris/HCl, 1 mM EDTA, pH 7.5).

### Methylation-specific PCR

The methylation status of the miR-663 promoter region was determined by methylation-specific PCR. Primers distinguishing unmethylated (U) and methylated (M) alleles were designed to amplify the sequence:

miR-663 M-forward: 5- GTTTTGTTTTTGAAGAAAAGAGGC −3;

miR-663 M-reverse: 5- CTACGTACGACAACCTTAAACGTT-3;

miR-663 U-forward: 5- GTTTTGTTTTTGAAGAAAAGAGGTG −3;

miR-663 U-reverse: 5- ACCTACATACAACAACCTTAAACATT −3.

Each PCR reaction contained 20 ng of sodium bisulphite-modified DNA, 250 pmol of each primer, 250 pmol deoxynucleoside triphosphate, 1 × PCR buffer, and one unit of ExTaq HS polymerase ( TAKARA Bio Inc. , Tokyo, Japan) in a final reaction volume of 20 μl. Cycling conditions were initial denaturation at 95°C for 3 min, 40 cycles of 94°C for 30 s, 58°C (M) or 56°C (U) for 30 s, and 72°C for 30 s. For each set of methylation-specific PCR reactions, in vitro-methylated genomic DNA treated with sodium bisulphite served as a positive methylation control. PCR products were separated on 4% agarose gels, stained with ethidium bromide and visualized under UV illumination. For cases with borderline results, PCR analyses were repeated.

### Bisulfite genomic sequencing

Bisulfite genomic sequencing (BGS) were performed as previously described [25]. BGS primers were from +86 to +384 including 21 CpGs miR-663 F:5- AGGTGTTTTGTTTTTGAAGAAAAGA −3 and miR-663 R:5- TACCCCAAAACACACCTCTTAAA −3. Amplified BGS products were TA-cloned , and five to six randomly chosen colonies were sequenced. DNA sequences were analyzed with BiQ Analyzer (http://biq-analyzer.bioinf.mpi-inf.mpg.de).

### Leukemia cell lines and primary cells treated with 5-aza-2′-deoxycytidine

De-methylation was induced with 5-aza-dC (5-Aza, Sigma-Aldrich, St Louis, MO, USA) treatment at a concentration that induced de-methylation of the DNA without killing the cells. Culture media for HL-60, MV4-11 and SHI-1 cells contained 5 μM 5-Aza, the primary leukemia cells were treated with 10 μM 5-Aza. DNA and RNA were extracted after 72 hours of 5-Aza treatment for the following analysis.

### Statistical analysis

SPSS v11.5 (SPSS Inc., Chicago, IL) was used for statistical analysis. Data are presented as means ± standard deviation. Group t-test was used to compare the expression of miR-663 between DMSO group and 5-Aza group. Statistical significance between methylated sample data and clinical pathological features of AML patients were analyzed by Pearson chi-square test or Fisher's exact test. Statistical significance of miR-663 expression among NBM and pediatric AML groups was determined using one-way ANOVA. A p < 0.05 was considered statistically significant.

## Results and discussion

### Analysis of CpG islands in the promoter of miR-663

**Figure 1:**
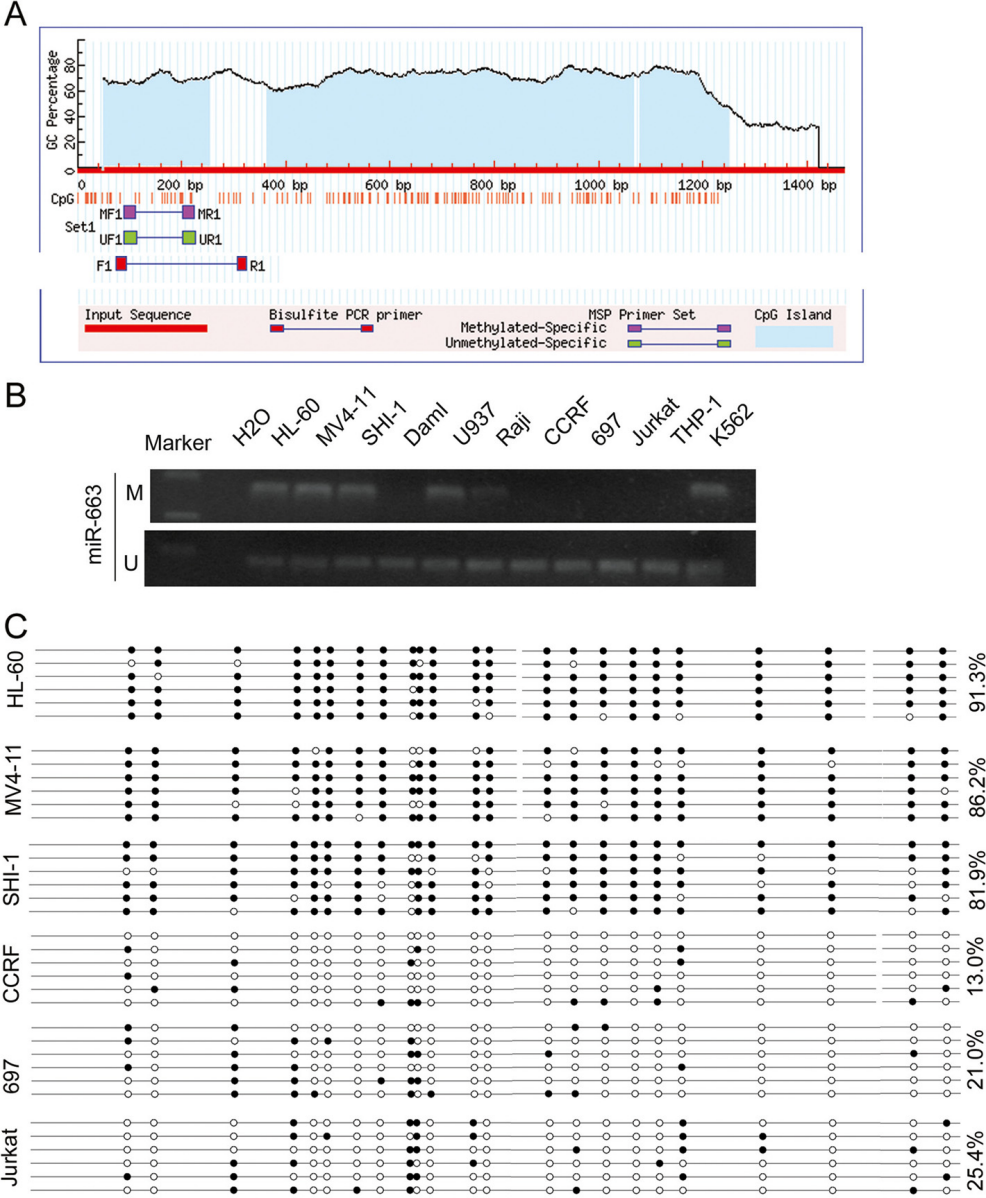
**The miR-663 promoter is methylated in AML cell lines. (A)** Three CpG island regions can be identified in the promoter of miR-663. **(B)** MSP analysis of the methylation status of miR-663 in leukemia cell lines shows that the promoter is hypermethylated in 5/11 cell lines. M and U represent MSP results using primer sets for methylated and unmethylated miR-663 genes, respectively. **(C)** BGS results show the rate of CpG methylation is 91.3% in HL-60 cells, 86.2% in MV4-11 cells, and 81.9% in SHI-1 cells compared to 13.0% in CCRF cells, 21.0% in 697 cells, and 25.4% in Jurkat cells. ● methylated cytosines; ○ unmethylated cytosines.

The correlation between aberrant methylation and downregulation of miR-663 has been extensively documented in numerous cancers and cell lines, including breast cancer and gastric cancer. These are discussed in the Background and Discussion. However, reports on the methylation status of miR-663 in the blood system, in particular in pediatric AML, are rare. Our analyses of promoter methylation of miRNAs in pediatric AML, using NimbleGen Human DNA Methylation 385 K Promoter Plus CpG Island Arrays, implied that the miR-663 promoter may be hypermethylated in AML (Additional file 1). Subsequent analyses of the miR-663 promoter sequence identified three CpG islands (Figure 1A). In summary, our results support our earlier supposition that miR-663 may act as a tumor suppressor in leukemia.

#### The miR-663 promoter is methylated in leukemia cell lines

Methylation-specific PCR (MSP) assays were performed to detect the methylation status of the miR-663 promoter in 11 leukemia cell lines. The MSP primer was designed using MethPrimer http://www.urogene.org/cgi-bin/methprimer/methprimer.cgi to encompass the CpG islands of the miR-663 promoter identified in Figure 1A. Our results showed that the miR-663 promoter was hypermethylated in five of the cell lines (HL-60, MV4-11, SHI-1, U937, and K562); and unmethylated in the remaining six cell lines (Dami, Raji, CCRF, Jurkat, 697, and THP-1). Figure 1B is a representative MSP result.

Bisulfite genomic sequencing (BGS) of the miR-663 promoter showed that the CpG islands were methylated in HL-60, MV4-11, and SHI-1 cell lines (91.3%, 86.2%, and 81.9%, respectively); whereas, they were almost unmethylated in CCRF, 697, and Jurkat cell lines (13.0%, 21.0%, and 25.4%, respectively). The BGS results are shown Figure 1C, and the sequences are given in Additional file 2. These results were consistent with the MSP assays.

**Figure 2:**
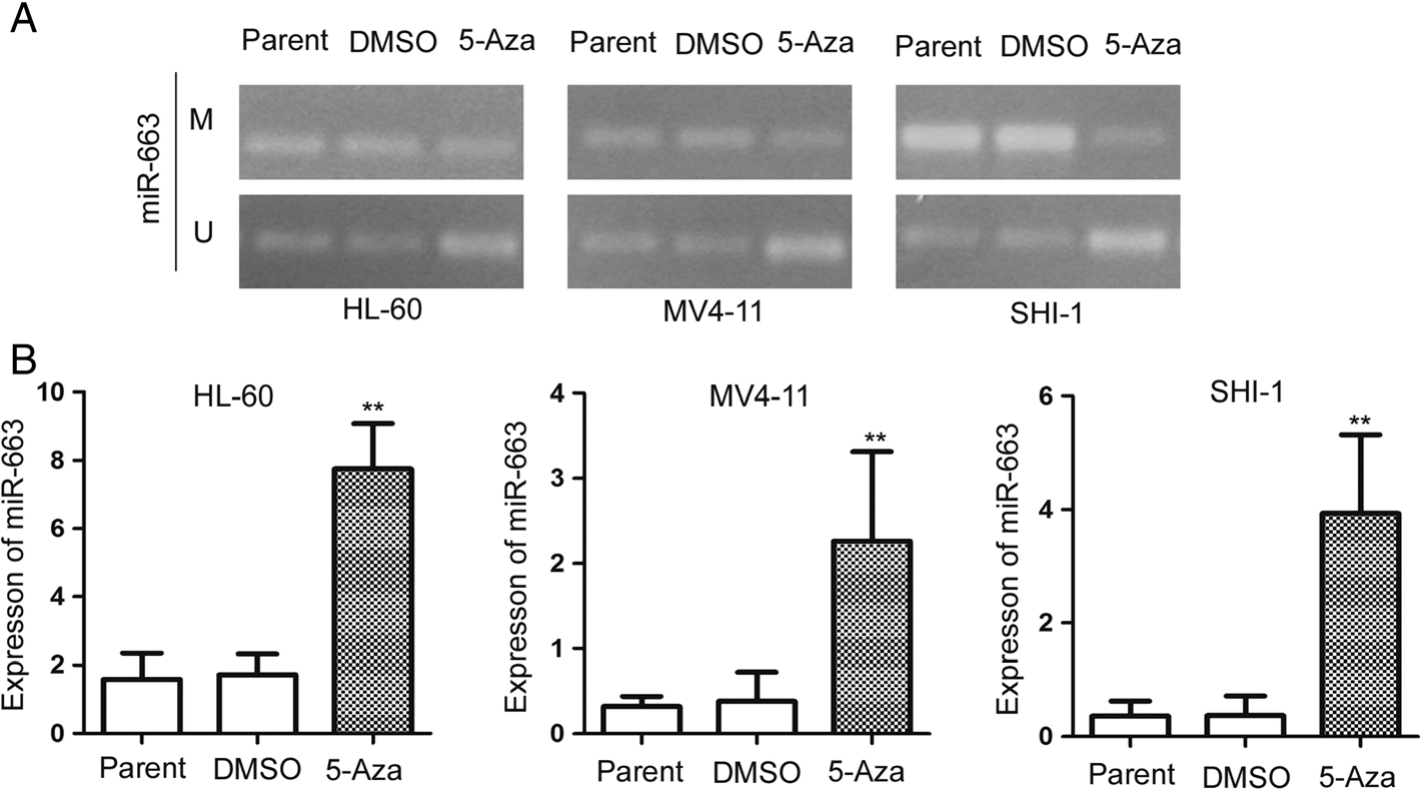
**MiR-663 is inactivated by promoter hypermethylation in AML cell lines. (A)** MSP analysis of the methylation status of miR-663 in leukemia cells lines 72 h after treatment with 5-Aza, or the same volume of DMSO solvent control, shows that miR-663 expression is significantly upregulated in HL-60, MV4-11, and SHI-1 cells. M and U represent MSP results using primer sets for methylated and unmethylated miR-663 genes, respectively. **(B)** The transcript level of miR-663 is upregulated in cells treated with 5-Aza compared to DMSO: 4.5-fold in HL-60 cells (5-Aza: 7.76 vs. DMSO: 1.72; *P* = 0.007); 5.9-fold in MV4-11 cells (5-Aza: 2.26 vs. DMSO: 0.38; *P* = 0.008); and 10.6-fold in SHI-1 cells (5-Aza: 3.93 vs. DMSO: 0.37; *P* = 0.004).

To confirm methylation of the miR-663 promoter, we treated the leukemia cell lines with the demethylation reagent 5-Aza. This is an epigenetic modifier that inhibits DNA methyltransferase activity through remodeling (opening) of chromatin, resulting in DNA demethylation (hypomethylation) and gene activation. Our results showed that 5-Aza treatment significantly upregulated miR-663 expression. As shown in Figure 2, miR-663 expression was upregulated 4.5-fold in HL-60 cells (5-Aza: 7.76 vs. DMSO: 1.72; *P* = 0.007); 5.9-fold in MV4-11 cells (5-Aza: 2.26 vs. DMSO: 0.38; *P* = 0.008); and 10.6-fold in SHI-1 cells (5-Aza: 3.93 vs. DMSO: 0.37; *P* = 0.004). These results were supported by the MSP analyses, which also showed a change in the methylation status of the miR-663 promoter after 5-Aza treatment.

In summary, these results showed that the miR-663 promoter was consistently significantly methylated in leukemia cells, such as HL-60, MV4-11, SHI-1, U937, and K562 human myeloid leukemia cell lines. In contrast the miR-663 promoter was unmethylated in human lymphoblastic leukemia cells, such as Jurkat, and 697. Based on these findings, we proposed that the promoter of miR-663 may be methylated in pediatric AML patients.

#### The miR-663 promoter is methylated in pediatric AML patients

**Figure 3:**
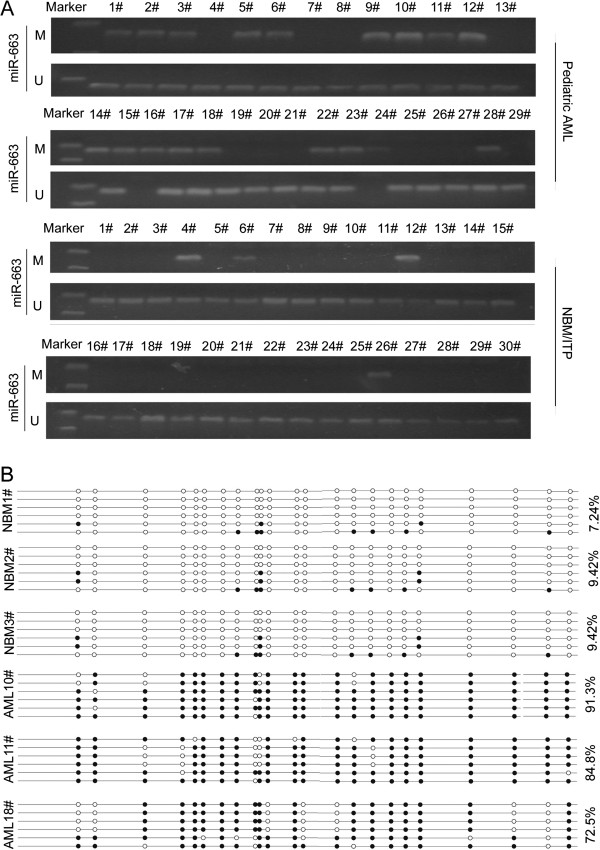
**The miR-663 promoter is methylated in pediatric AML. (A)** MSP analysis of the methylation status of miR-663 shows aberrant methylation in pediatric AML samples compared to NBM/ITP control samples. M and U represent MSP results using primer sets for methylated and unmethylated miR-663 genes, respectively. **(B)** BGS results of three AML samples and three NBM samples show that CpGs are methylated in the AML samples compared to NBM control samples (91.3%, 84.8%, and 72.5% in AML10#, AML11#, and AML18#, respectively; 7.24%, 9.42%, and 9.42% in NBM1#, NBM2#, and NBM3#, respectively). ● methylated cytosines; ○ unmethylated cytosines.

We next examined the methylation status of the miR-663 promoter in pediatric AML samples and NBM/ITP (normal bone marrow/idiopathicthrombocytopenic purpura) control samples. Aberrant methylation of miR-663 was observed 10% (3/30) of the NBM control samples compared to 41.4% (29/70) of the pediatric AML samples (Figure 3A).

Three NBM samples and three AML samples were further analyzed by BGS (Figure 3B). The results showed that the CpG islands in the miR-663 promoter were methylated in the AML samples (91.3%, 84.8%, and 72.5% in AML10#, AML11#, and AML18#, respectively). In contrast, the CpG islands in the miR-663 promoter were almost unmethylated in the NBM samples (7.24%, 9.42%, and 9.42 in NBM1#, NBM2#, and NBM3#, respectively). These results were supported by MSP assays.

**Figure 4:**
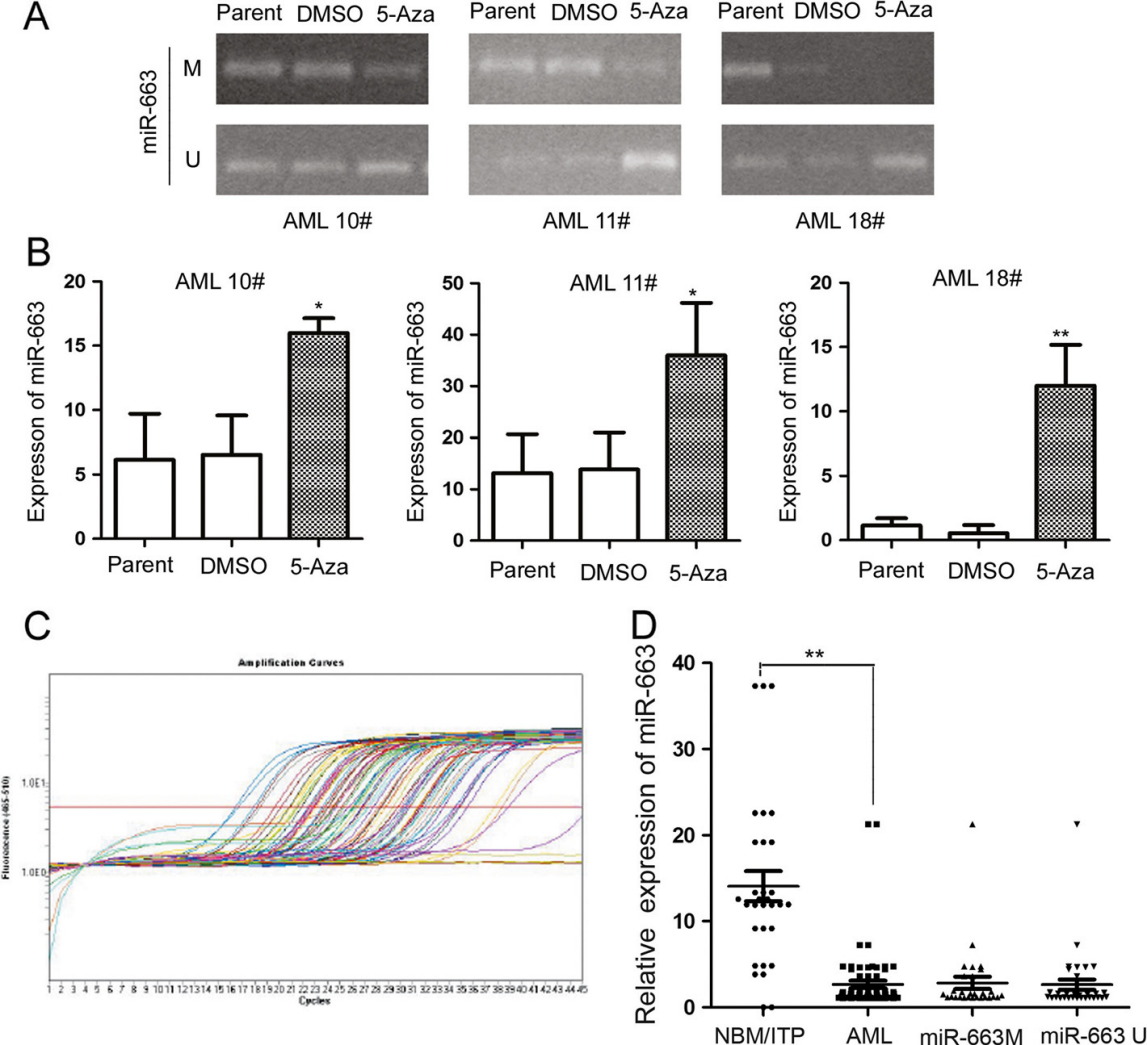
**MiR-663 is inactivated by promoter hypermethylation in pediatric AML. (A)** MSP analysis of the methylation status of miR-663 in leukemia primary cells 72 h after treatment with 5-Aza, or the same volume of DMSO, shows that the methylation status of the miR-663 promoter is altered in the three primary AML samples. M and U represent MSP results using primer sets for methylated and unmethylated miR-663 genes, respectively. **(B)** The transcript level of miR-663 is upregulated in primary AML cells treated with 5-Aza compared to DMSO control samples: 2.44-fold in AML10# (5-Aza: 15.9 vs. DMSO: 6.54; *P* = 0.02); 2.6-fold in AML11# (5-Aza: 35.99 vs. DMSO: 13.9; *P* = 0.04); and 22.2-fold in AML18# (5-Aza: 11.9 vs. DMSO: 0.54; *P* < 0.01). **(C)** Real-time analysis of miR-663 transcript expression in pediatric AML patients shows that patients with miR-663 methylation (*n* = 29) and those without miR-663 methylation (*n* = 41) both have significantly lower miR-663 transcript expression levels compared to controls. **(D)** The transcript level of the miR-663 gene in 70 AML patients is 2.67 ± 3.55 compared to 14.06 ± 9.81 in 30 control samples (*P* < 0.001).

We confirmed methylation of the miR-663 promoter by showing that miR-663 expression was significantly upregulated when these leukemia primary cells were treated with 5-Aza (Figure 4B): miR-663 was upregulated 2.44-fold in AML10# (5-Aza: 15.9 vs. DMSO: 6.54; *P* = 0.02); 2.6-fold in AML11# (5-Aza: 35.99 vs. DMSO: 13.9; *P* = 0.04); and 22.2-fold in AML18# (5-Aza: 11.9 vs. DMSO: 0.54; *P* < 0.01). MSP analysis supported these results by showing the methylation status of the miR-663 promoter was changed after 5-Aza treatment (Figure 4A).

Table 1 shows there were no significant differences in clinical features, such as sex, age, initial hemoglobin level, white blood cell counts, platelet counts, and chromosomal abnormalities between patients with and without methylated miR-663.

#### Expression of the miR-663 transcript in pediatric AML patients

The transcript levels of miR-663 were examined in 70 pediatric AML patients by real-time PCR. As shown in Table 1, miR-663 expression was significantly decreased in AML patients (2.67 ± 3.55; *P* < 0.001) compared to 30 NBM/ITP controls (14.06 ± 9.81); however, patients with methylated miR-663 (*n* = 29) showed similar miR-663 transcript levels compared to those without methylated miR-663 (*n* = 41; *P* = 0.827; Figure 4C); and both those AML patients with and without methylated miR-663 showed significantly lower miR-663 transcript levels compared to controls (*P* < 0.001; Figure 4D). This implies that other mechanisms may be involved in the downregulation of miR-663 in pediatric AML, such as other post-transcriptional modifications, gene deletions, copy number reductions, and histone code modifications; therefore, further research focusing on the mechanism of miR-663 downregulation in pediatric leukemia is required.

MiR-663 appears to have multiple functions that vary between different experimental models [14,15,18,22,24]. Instances of miR-663 hypermethylation and loss of function have been documented in numerous cancers. For example, a miRNA microarray study by Yi *et al*. reported upregulation of miR-663 in nasopharyngeal carcinoma (NPC) cells compared to human immortalized nasopharyngeal epithelium cells. This was confirmed in tissue samples from patients diagnosed and treated with NPC [24]. Inhibition of miR-663 impaired the proliferation of NPC cells in vitro and NPC tumor xenograft growth in nude mice. Yang *et al*. showed that miR-663 expression was upregulated in K562 cells after 5-Aza treatment, but that miR-663 expression levels were lower in K562, U937, Kasumi cell lines and newly diagnosed patients, compared to healthy subjects [26]. This was supported by an MSP study which found that the CpG islands of miR-663 were methylated in K562 cell lines [27]. Overexpression of miR-663 can suppress the proliferation of K562 cells, indicating that miR-663 may also exert a suppressive effect in leukemia cells [27]. In addition, the miR-663 gene has been reported to be hypermethylated in breast cancer [17].

To our knowledge, this is the first report describing the expression of miR-663 and promoter methylation status in childhood leukemia. In this study, hypermethylation of the miR-663 promoter was detected in 45.5% (5/11) of the leukemia cell lines and in 41.4% of the pediatric AML primary tumor cells, but not in normal control cells. Although promoter hypermethylation of miR-663 was found at a high frequency in AML samples, it was not associated with the sex, age or initial hemoglobin levels of the patients.

In summary, our results show that the expression of miR-663 was significantly lower in pediatric AML compared to NBM control samples; hypermethylation of the miR-663 promoter was observed at a high frequency in both AML cell lines and pediatric AML samples; and miR-663 inactivation by hypermethylation of the promoter could be affected by 5-Aza demethylation.

## Conclusions

By demonstrating that epigenetic inactivation of miR-663 by promoter hypermethylation can be observed in both AML cell lines and pediatric AML samples, our study suggests that miR-663 may be considered as a putative tumor suppressor gene in pediatric AML. In addition, our findings imply that transcriptional silencing of the miR-663 gene might be involved in the tumorigenesis of pediatric AML, and that promoter hypermethylation of miR-663 might be an early event in the development of pediatric AML. However, further research focusing on the function and the mechanism of miR-663 in pediatric leukemia is required.

## Competing interests

The authors have no conflicts of interest to disclose.

## Authors’ contributions

PJ designed and directed the study. TYF and NJ finished the most of the experiments. WJ and FX coordinated data collection and quality control, and assisted in the interpretation of results. WN, ZWL, WD, PL and LJ participated in acquiring laboratory data analysis. NJ and XPF participated in study design and coordination, data analysis and interpretation and drafted the manuscript. All authors read and approved the final manuscript.

## Pre-publication history

The pre-publication history for this paper can be accessed here:

http://www.biomedcentral.com/1471-2350/14/74/prepub

## Supplementary Material

Additional file 1 **Analysis of promoter methylation of miRNAs in pediatric AML using NimbleGen Human DNA Methylation 385 K Promoter Plus CpG Island Arrays.** We had previously analyzed the methylation status of 63 miRNAs in four pediatric AML samples (M1, M2, M3, and M4) and three NBM samples (C1, C2, and C3) using NimbleGen Human DNA Methylation Arrays. Each red box represents the number of methylation peaks (PeakScore) overlapping the promoter region for the corresponding miRNA. The PeakScore is defined as the average -log10 (*P*-value) from probes within the peak. The scores reflect the probability of positive methylation enrichment. The DNA methylation array analysis shows that the promoter of miR-663 is significantly methylated in AML samples (4/4), and unmethylated in NBM samples (0/3). Click here for file

Additional file 2 **Bisulfite genomic sequencing of leukemia cells.** The bisulfite genomic sequencing (BGS) primers (from +86 to +384) include 21 CpGs. The amplified BGS products were TA-cloned and five to six randomly chosen colonies were sequenced. The DNA sequences of six leukemia cells are presented. Click here for file
