# Supplementary figures and images for: The promoter of miR-663 is hypermethylated in Chinese pediatric acute myeloid leukemia (AML)
Source: BMC Med Genet. 2013 Jul 19;14:74. doi: 10.1186/1471-2350-14-74 (PMC3726388; doi:10.1186/1471-2350-14-74)

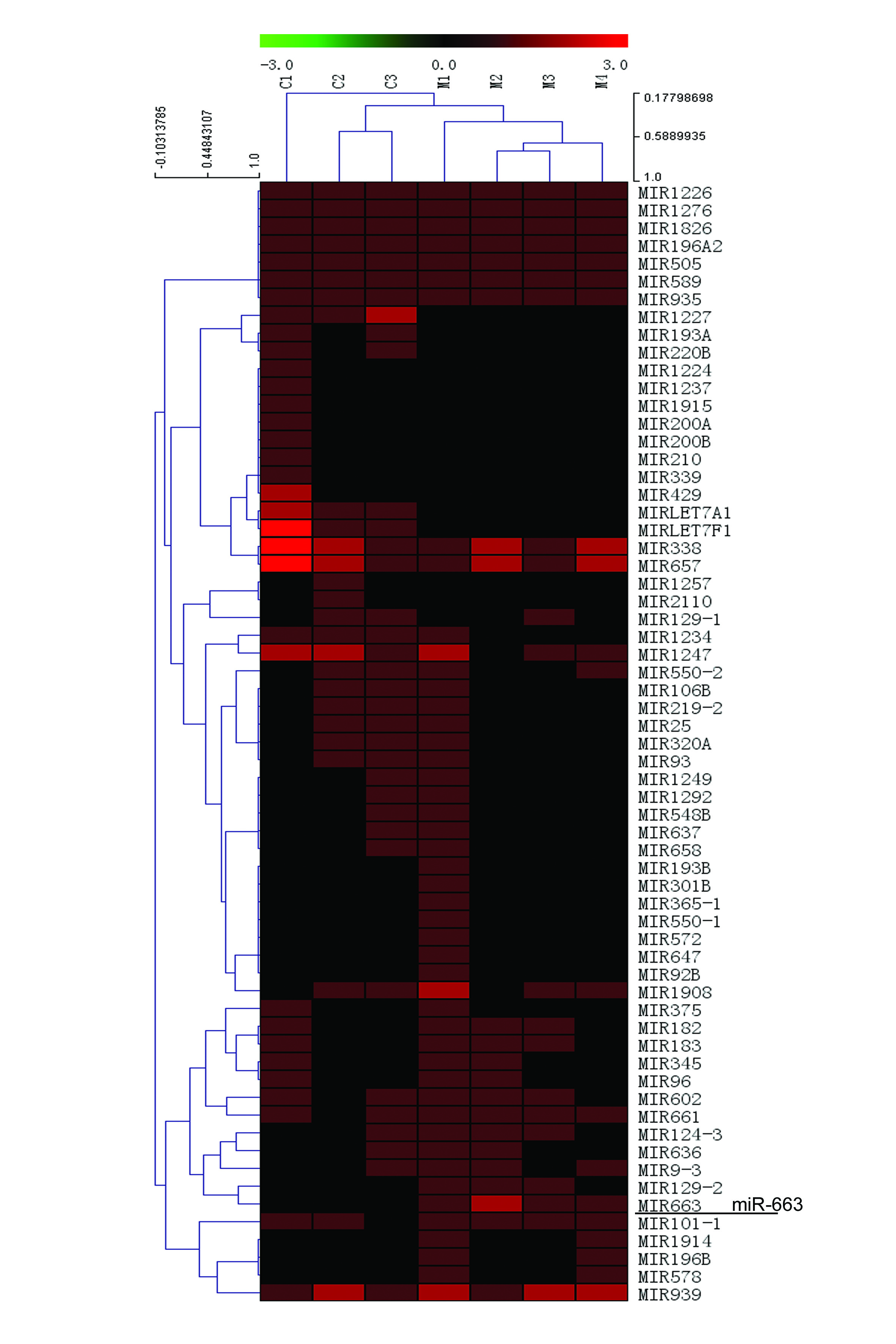

Supplement: Additional file 1 — Analysis of promoter methylation of miRNAs in pediatric AML using NimbleGen Human DNA Methylation 385 K Promoter Plus CpG Island Arrays. We had previously analyzed the methylation status of 63 miRNAs in four pediatric AML samples (M1, M2, M3, and M4) and three NBM samples (C1, C2, and C3) using NimbleGen Human DNA Methylation Arrays. Each red box represents the number of methylation peaks (PeakScore) overlapping the promoter region for the corresponding miRNA. The PeakScore is defined as the average -log10 (P-value) from probes within the peak. The scores reflect the probability of positive methylation enrichment. The DNA methylation array analysis shows that the promoter of miR-663 is significantly methylated in AML samples (4/4), and unmethylated in NBM samples (0/3). [file 1471-2350-14-74-S1.jpeg]
