# Supplementary material for: The promoter of miR-663 is hypermethylated in Chinese pediatric acute myeloid leukemia (AML)
Source: BMC Med Genet. 2013 Jul 19;14:74. doi: 10.1186/1471-2350-14-74 (PMC3726388; doi:10.1186/1471-2350-14-74)
Supplement: Additional file 2 — Bisulfite genomic sequencing of leukemia cells. The bisulfite genomic sequencing (BGS) primers (from +86 to +384) include 21 CpGs. The amplified BGS products were TA-cloned and five to six randomly chosen colonies were sequenced. The DNA sequences of six leukemia cells are presented. [file 1471-2350-14-74-S2.doc]

**Bisulfite genomic sequence of HL-60 cells**

1#

AGGTGTTTTGTTTTTGAAGAAAAGAGGCGGGTGGACGGGGTGGGGGGGGGGGTGGGAGGCGGGGGTTTGTGGAGTTGCGTTTACGTTCGTATTTGTCGGTTTTCGTTTTGATCGCGAACGTTTAAGGTTGTCGTACGTAGGTTTTTTTTGGTATTATAGGTTTTTTTTTTTTTTTAGGTTTTTTTGAGCGTTTTTGCGGGTTTTTCGGGTTTTACGAGGGGCGGTTGGCGGGTGGGGAGTGTGATTTATTTTCGGTGAGAAAGTTTTTTTTAGCG

ATTTAAGAGGTGTGTTTTGGGGTACGGGATTTTTCGGTTT

2#

AGGTGTTTTGTTTTTGAAGAAAAGAGGTGGGTGGACGGGGTGGGGGGGGGGGTGGGAGGTGGGGGTTTGTGGAGTTGCGTTTACGTTCGTATTTGTCGGTTTTCGTTTTGATCGTGAACGTTTAAGGTTGTCGTACGTAGGTTTTTTTTGGTATTATAGGTTTTTTTTTTTTTTTAGGTTTTTTTGAGCGTTTTTGTGGGTTTTTCGGGTTTTACGAGGGGCGGTTGGCGGGTGGGGAGTGTGATTTATTTTCGGTGAGAAAGTTTTTTTTAGCG

ATTTAAGAGGTGTGTTTTGGGGTACGGGATTTTTCGGTTT

3#

AGGTGTTTTGTTTTTGAAGAAAAGAGGCGGGTGGATGGGGTGGGGGGGGGGGTGGGAGGCGGGGGTTTGTGGAGTTGCGTTTACGTTCGTATTTGTCGGTTTTCGTTTTGATCGCGAACGTTTAAGGTTGTCGTACGTAGGTTTTTTTTGGTATTATAGGTTTTTTTTTTTTTTTAGGTTTTTTTGAGCGTTTTTGCGGGTTTTTCGGGTTTTACGAGGGGCGGTTGGCGGGTGGGGAGTGTGATTTATTTTCGGTGAGAAAGTTTTTTTTAGCG

ATTTAAGAGGTGTGTTTTGGGGTACGGGATTTTTCGGTTT

4#

AGGTGTTTTGTTTTTGAAGAAAAGAGGCGGGTGGACGGGGTGGGGGGGGGGGTGGGAGGCGGGGGTTTGTGGAGTTGCGTTTACGTTCGTATTTGTCGGTTTTCGTTTTGATTGCGAACGTTTAAGGTTGTCGTACGTAGGTTTTTTTTGGTATTATAGGTTTTTTTTTTTTTTTAGGTTTTTTTGAGCGTTTTTGCGGGTTTTTCGGGTTTTACGAGGGGCGGTTGGCGGGTGGGGAGTGTGATTTATTTTCGGTGAGAAAGTTTTTTTTAGCG

ATTTAAGAGGTGTGTTTTGGGGTACGGGATTTTTCGGTTT

5#

AGGTGTTTTGTTTTTGAAGAAAAGAGGCGGGTGGACGGGGTGGGGGGGGGGGTGGGAGGCGGGGGTTTGTGGAGTTGCGTTTACGTTCGTATTTGTCGGTTTTCGTTTTGATCGCGAACGTTTAAGGTTGTTGTACGTAGGTTTTTTTTGGTATTATAGGTTTTTTTTTTTTTTTAGGTTTTTTTGAGCGTTTTTGCGGGTTTTTCGGGTTTTACGAGGGGCGGTTGGCGGGTGGGGAGTGTGATTTATTTTCGGTGAGAAAGTTTTTTTTAGCG

ATTTAAGAGGTGTGTTTTGGGGTACGGGATTTTTCGGTTT

6#

AGGTGTTTTGTTTTTGAAGAAAAGAGGCGGGTGGACGGGGTGGGGGGGGGGGTGGGAGGCGGGGGTTTGTGGAGTTGCGTTTACGTTCGTATTTGTCGGTTTTCGTTTTGATTGCGAACGTTTAAGGTTGTCGTATGTAGGTTTTTTTTGGTATTATAGGTTTTTTTTTTTTTTTAGGTTTTTTTGAGCGTTTTTGCGGGTTTTTTGGGTTTTACGAGGGGCGGTTGGTGGGTGGGGAGTGTGATTTATTTTCGGTGAGAAAGTTTTTTTTAGCG

ATTTAAGAGGTGTGTTTTGGGGTATGGGATTTTTCGGTTT

**Bisulfite genomic sequence of MV4-11 cells**

1#

AGGTGTTTTGTTTTTGAAGAAAAGAGGCGGGTGGACGGGGTGGGGGGGGGGGTGGGAGGCGGGGGTTTGTGGAGTTGCGTTTATGTTCGTATTTGTCGGTTTTCGTTTTGATTGTGAACGTTTAAGGTTGTTGTACGTAGGTTTTTTTTGGTATTATAGGTTTTTTTTTTTTTTTAGGTTTTTTTGAGCGTTTTTGTGGGTTTTTCGGGTTTTACGAGGGGCGGTTGGCGGGTGGGGAGTGTGATTTATTTTCGGTGAGAAAGTTTTTTTTAGCG

ATTTAAGAGGTGTGTTTTGGGGTACGGGATTTTTCGGTTT

2#

AGGTGTTTTGTTTTTGAAGAAAAGAGGCGGGTGGACGGGGTGGGGGGGGGGGTGGGAGGCGGGGGTTTGTGGAGTTGCGTTTACGTTCGTATTTGTCGGTTTTCGTTTTGATTGCGAACGTTTAAGGTTGTCGTACGTAGGTTTTTTTTGGTATTATAGGTTTTTTTTTTTTTTTAGGTTTTTTTGAGTGTTTTTGCGGGTTTTTCGGGTTTTACGAGGGGTGGTTGGTGGGTGGGGAGTGTGATTTATTTTCGGTGAGAAAGTTTTTTTTAGTG

ATTTAAGAGGTGTGTTTTGGGGTACGGGATTTTTCGGTTT

3#

AGGTGTTTTGTTTTTGAAGAAAAGAGGCGGGTGGACGGGGTGGGGGGGGGGGTGGGAGGCGGGGGTTTGTGGAGTTGCGTTTACGTTCGTATTTGTCGGTTTTCGTTTTGATCGCGAACGTTTAAGGTTGTCGTACGTAGGTTTTTTTTGGTATTATAGGTTTTTTTTTTTTTTTAGGTTTTTTTGAGCGTTTTTGCGGGTTTTTCGGGTTTTACGAGGGGCGGTTGGCGGGTGGGGAGTGTGATTTATTTTCGGTGAGAAAGTTTTTTTTAGCG

ATTTAAGAGGTGTGTTTTGGGGTACGGGATTTTTCGGTTT

4#

AGGTGTTTTGTTTTTGAAGAAAAGAGGCGGGTGGACGGGGTGGGGGGGGGGGTGGGAGGCGGGGGTTTGTGGAGTTGTGTTTACGTTCGTATTTGTCGGTTTTCGTTTTGATCGCGAACGTTTAAGGTTGTCGTACGTAGGTTTTTTTTGGTATTATAGGTTTTTTTTTTTTTTTAGGTTTTTTTGAGCGTTTTTGCGGGTTTTTCGGGTTTTACGAGGGGCGGTTGGCGGGTGGGGAGTGTGATTTATTTTCGGTGAGAAAGTTTTTTTTAGCG

ATTTAAGAGGTGTGTTTTGGGGTACGGGATTTTTTGGTTT

5#

AGGTGTTTTGTTTTTGAAGAAAAGAGGCGGGTGGACGGGGTGGGGGGGGGGGTGGGAGGTGGGGGTTTGTGGAGTTGTGTTTACGTTCGTATTTGTCGGTTTTCGTTTTGATTGTGAACGTTTAAGGTTGTCGTACGTAGGTTTTTTTTGGTATTATAGGTTTTTTTTTTTTTTTAGGTTTTTTTGAGCGTTTTTGCGGGTTTTTTGGGTTTTACGAGGGGCGGTTGGCGGGTGGGGAGTGTGATTTATTTTCGGTGAGAAAGTTTTTTTTAGCG

ATTTAAGAGGTGTGTTTTGGGGTACGGGATTTTTCGGTTT

6#

AGGTGTTTTGTTTTTGAAGAAAAGAGGCGGGTGGACGGGGTGGGGGGGGGGGTGGGAGGCGGGGGTTTGTGGAGTTGCGTTTACGTTCGTATTTGTTGGTTTTCGTTTTGATCGCGAACGTTTAAGGTTGTCGTACGTAGGTTTTTTTTGGTATTATAGGTTTTTTTTTTTTTTTAGGTTTTTTTGAGTGTTTTTGCGGGTTTTTCGGGTTTTACGAGGGGCGGTTGGCGGGTGGGGAGTGTGATTTATTTTCGGTGAGAAAGTTTTTTTTAGCG

ATTTAAGAGGTGTGTTTTGGGGTACGGGATTTTTCGGTTT

**Bisulfite genomic sequence of SHI-1 cells**

1#

AGGTGTTTTGTTTTTGAAGAAAAGAGGCGGGTGGACGGGGTGGGGGGGGGGGTGGGAGGCGGGGGTTTGTGGAGTTGCGTTTACGTTCGTATTTGTCGGTTTTCGTTTTGATCGCGAACGTTTAAGGTTGTCGTACGTAGGTTTTTTTTGGTATTATAGGTTTTTTTTTTTTTTTAGGTTTTTTTGAGCGTTTTTGCGGGTTTTTCGGGTTTTACGAGGGGCGGTTGGCGGGTGGGGAGTGTGATTTATTTTCGGTGAGAAAGTTTTTTTTAGCG

ATTTAAGAGGTGTGTTTTGGGGTACGGGATTTTTCGGTTT

2#

AGGTGTTTTGTTTTTGAAGAAAAGAGGCGGGTGGACGGGGTGGGGGGGGGGGTGGGAGGCGGGGGTTTGTGGAGTTGCGTTTACGTTCGTATTTGTCGGTTTTCGTTTTGATTGTGAACGTTTAAGGTTGTTGTACGTAGGTTTTTTTTGGTATTATAGGTTTTTTTTTTTTTTTAGGTTTTTTTGAGCGTTTTTGCGGGTTTTTCGGGTTTTACGAGGGGCGGTTGGTGGGTGGGGAGTGTGATTTATTTTTGGTGAGAAAGTTTTTTTTAGCG

ATTTAAGAGGTGTGTTTTGGGGTACGGGATTTTTCGGTTT

3#

AGGTGTTTTGTTTTTGAAGAAAAGAGGTGGGTGGATGGGGTGGGGGGGGGGGTGGGAGGCGGGGGTTTGTGGAGTTGCGTTTACGTTCGTATTTGTCGGTTTTCGTTTTGATCGCGAATGTTTAAGGTTGTCGTATGTAGGTTTTTTTTGGTATTATAGGTTTTTTTTTTTTTTTAGGTTTTTTTGAGTGTTTTTGCGGGTTTTTCGGGTTTTACGAGGGGCGGTTGGCGGGTGGGGAGTGTGATTTATTTTCGGTGAGAAAGTTTTTTTTAGTG

ATTTAAGAGGTGTGTTTTGGGGTATGGGATTTTTCGGTTT

4#

AGGTGTTTTGTTTTTGAAGAAAAGAGGCGGGTGGACGGGGTGGGGGGGGGGGTGGGAGGCGGGGGTTTGTGGAGTTGCGTTTACGTTTGTATTTGTCGGTTTTTGTTTTGATTGCGAACGTTTAAGGTTGTCGTATGTAGGTTTTTTTTGGTATTATAGGTTTTTTTTTTTTTTTAGGTTTTTTTGAGCGTTTTTGCGGGTTTTTCGGGTTTTACGAGGGGCGGTTGGCGGGTGGGGAGTGTGATTTATTTTTGGTGAGAAAGTTTTTTTTAGCG

ATTTAAGAGGTGTGTTTTGGGGTATGGGATTTTTCGGTTT

5#

AGGTGTTTTGTTTTTGAAGAAAAGAGGCGGGTGGACGGGGTGGGGGGGGGGGTGGGAGGCGGGGGTTTGTGGAGTTGTGTTTACGTTCGTATTTGTCGGTTTTTGTTTTGATTGCGAACGTTTAAGGTTGTCGTACGTAGGTTTTTTTTGGTATTATAGGTTTTTTTTTTTTTTTAGGTTTTTTTGAGCGTTTTTGCGGGTTTTTCGGGTTTTACGAGGGGCGGTTGGTGGGTGGGGAGTGTGATTTATTTTCGGTGAGAAAGTTTTTTTTAGCG

ATTTAAGAGGTGTGTTTTGGGGTACGGGATTTTTTGGTTT

6#

AGGTGTTTTGTTTTTGAAGAAAAGAGGCGGGTGGACGGGGTGGGGGGGGGGGTGGGAGGTGGGGGTTTGTGGAGTTGCGTTTACGTTCGTATTTGTCGGTTTTCGTTTTGATTGCGAACGTTTAAGGTTGTCGTACGTAGGTTTTTTTTGGTATTATAGGTTTTTTTTTTTTTTTAGGTTTTTTTGAGCGTTTTTGTGGGTTTTTCGGGTTTTACGAGGGGCGGTTGGCGGGTGGGGAGTGTGATTTATTTTCGGTGAGAAAGTTTTTTTTAGCG

ATTTAAGAGGTGTGTTTTGGGGTATGGGATTTTTCGGTTT

**Bisulfite genomic sequence of CCRF cells**

1#

AGGTGTTTTGTTTTTGAAGAAAAGAGGTGGGTGGATGGGGTGGGGGGGGGGGTGGGAGGTGGGGGTTTGTGGAGTTGTGTTTATGTTTGTATTTGTTGGTTTTTGTTTTGATTGTGAATGTTTAAGGTTGTTGTATGTAGGTTTTTTTTGGTATTATAGGTTTTTTTTTTTTTTTAGGTTTTTTTGAGTGTTTTTGTGGGTTTTTTGGGTTTTATGAGGGGTGGTTGGTGGGTGGGGAGTGTGATTTATTTTTGGTGAGAAAGTTTTTTTTAGTG

ATTTAAGAGGTGTGTTTTGGGGTATGGGATTTTTTGGTTT

2#

AGGTGTTTTGTTTTTGAAGAAAAGAGGCGGGTGGATGGGGTGGGGGGGGGGGTGGGAGGTGGGGGTTTGTGGAGTTGTGTTTATGTTTGTATTTGTTGGTTTTTGTTTTGATTGCGAATGTTTAAGGTTGTTGTATGTAGGTTTTTTTTGGTATTATAGGTTTTTTTTTTTTTTTAGGTTTTTTTGAGTGTTTTTGTGGGTTTTTTGGGTTTTATGAGGGGTGGTTGGCGGGTGGGGAGTGTGATTTATTTTTGGTGAGAAAGTTTTTTTTAGTG

ATTTAAGAGGTGTGTTTTGGGGTATGGGATTTTTTGGTTT

3#

AGGTGTTTTGTTTTTGAAGAAAAGAGGTGGGTGGATGGGGTGGGGGGGGGGGTGGGAGGCGGGGGTTTGTGGAGTTGTGTTTATGTTTGTATTTGTTGGTTTTTGTTTTGATCGTGAATGTTTAAGGTTGTTGTATGTAGGTTTTTTTTGGTATTATAGGTTTTTTTTTTTTTTTAGGTTTTTTTGAGTGTTTTTGTGGGTTTTTTGGGTTTTATGAGGGGTGGTTGGCGGGTGGGGAGTGTGATTTATTTTTGGTGAGAAAGTTTTTTTTAGTG

ATTTAAGAGGTGTGTTTTGGGGTATGGGATTTTTTGGTTT

4#

AGGTGTTTTGTTTTTGAAGAAAAGAGGCGGGTGGATGGGGTGGGGGGGGGGGTGGGAGGTGGGGGTTTGTGGAGTTGTGTTTATGTTTGTATTTGTTGGTTTTTGTTTTGATTGTGAATGTTTAAGGTTGTTGTATGTAGGTTTTTTTTGGTATTATAGGTTTTTTTTTTTTTTTAGGTTTTTTTGAGTGTTTTTGTGGGTTTTTTGGGTTTTATGAGGGGTGGTTGGTGGGTGGGGAGTGTGATTTATTTTTGGTGAGAAAGTTTTTTTTAGTG

ATTTAAGAGGTGTGTTTTGGGGTATGGGATTTTTTGGTTT

5#

AGGTGTTTTGTTTTTGAAGAAAAGAGGTGGGTGGACGGGGTGGGGGGGGGGGTGGGAGGCGGGGGTTTGTGGAGTCGTGTTTATGTTTGTATTTGTTGGTTTTTGTTTTGATTGTGAATGTTTAAGGTTGTTGTATGTAGGTTTTTTTTGGTATTATAGGTTTTTTTTTTTTTTTAGGTTTTTTTGAGTGTTTTTGTGGGTTTTTTGGGTTTTATGAGGGGCGGTTGGTGGGTGGGGAGTGTGATTTATTTTTGGTGAGAAAGTTTTTTTTAGTG

ATTTAAGAGGTGTGTTTTGGGGTATGGGATTTTTCGGTTT

6#

AGGTGTTTTGTTTTTGAAGAAAAGAGGTGGGTGGATGGGGTGGGGGGGGGGGTGGGAGGTGGGGGTTTGTGGAGTTGTGTTTATGTTTGTATTTGTTGGTTTTCGTTTTGATCGCGAATGTTTAAGGTTGTTGTATGTAGGTTTTTTTTGGTATTATAGGTTTTTTTTTTTTTTTAGGTTTTTTTGAGTGTTTTTGCGGGTTTTTCGGGTTTTATGAGGGGCGGTTGGTGGGTGGGGAGTGTGATTTATTTTTGGTGAGAAAGTTTTTTTTAGTG

ATTTAAGAGGTGTGTTTTGGGGTACGGGATTTTTTGGTTT

**Bisulfite genomic sequence of 697 cells**

1#

AGGTGTTTTGTTTTTGAAGAAAAGAGGCGGGTGGATGGGGTGGGGGGGGGGGTGGGAGGCGGGGGTTTGTGGAGTTGTGTTTATGTTTGTATTTGTTGGTTTTTGTTTTGATTGTGAATGTTTAAGGTTGTTGTATGTAGGTTTTTTTTGGTATTATAGGTTTTTTTTTTTTTTTAGGTTTTTTTGAGTGTTTTTGCGGGTTTTTCGGGTTTTATGAGGGGTGGTTGGTGGGTGGGGAGTGTGATTTATTTTTGGTGAGAAAGTTTTTTTTAGTG

ATTTAAGAGGTGTGTTTTGGGGTATGGGATTTTTTGGTTT

2#

AGGTGTTTTGTTTTTGAAGAAAAGAGGCGGGTGGATGGGGTGGGGGGGGGGGTGGGAGGTGGGGGTTTGTGGAGTTGCGTTTATGTTCGTATTTGTTGGTTTTTGTTTTGATCGTGAATGTTTAAGGTTGTTGTATGTAGGTTTTTTTTGGTATTATAGGTTTTTTTTTTTTTTTAGGTTTTTTTGAGTGTTTTTGTGGGTTTTTTGGGTTTTATGAGGGGTGGTTGGTGGGTGGGGAGTGTGATTTATTTTTGGTGAGAAAGTTTTTTTTAGTG

ATTTAAGAGGTGTGTTTTGGGGTATGGGATTTTTTGGTTT

3#

AGGTGTTTTGTTTTTGAAGAAAAGAGGTGGGTGGATGGGGTGGGGGGGGGGGTGGGAGGCGGGGGTTTGTGGAGTTGTGTTTATGTTTGTATTTGTTGGTTTTTGTTTTGATCGCGAATGTTTAAGGTTGTTGTATGTAGGTTTTTTTTGGTATTATAGGTTTTTTTTTTTTTTTAGGTTTTTTTGAGCGTTTTTGTGGGTTTTTTGGGTTTTATGAGGGGTGGTTGGTGGGTGGGGAGTGTGATTTATTTTTGGTGAGAAAGTTTTTTTTAGTG

ATTTAAGAGGTGTGTTTTGGGGTACGGGATTTTTTGGTTT

4#

AGGTGTTTTGTTTTTGAAGAAAAGAGGCGGGTGGATGGGGTGGGGGGGGGGGTGGGAGGCGGGGGTTTGTGGAGTTGCGTTTATGTTTGTATTTGTTGGTTTTTGTTTTGATTGTGAATGTTTAAGGTTGTTGTATGTAGGTTTTTTTTGGTATTATAGGTTTTTTTTTTTTTTTAGGTTTTTTTGAGTGTTTTTGTGGGTTTTTTGGGTTTTATGAGGGGTGGTTGGCGGGTGGGGAGTGTGATTTATTTTTGGTGAGAAAGTTTTTTTTAGTG

ATTTAAGAGGTGTGTTTTGGGGTATGGGATTTTTTGGTTT

5#

AGGTGTTTTGTTTTTGAAGAAAAGAGGTGGGTGGATGGGGTGGGGGGGGGGGTGGGAGGCGGGGGTTTGTGGAGTTGCGTTTATGTTTGTATTTGTTGGTTTTCGTTTTGATCGCGAATGTTTAAGGTTGTTGTATGTAGGTTTTTTTTGGTATTATAGGTTTTTTTTTTTTTTTAGGTTTTTTTGAGTGTTTTTGTGGGTTTTTTGGGTTTTATGAGGGGTGGTTGGTGGGTGGGGAGTGTGATTTATTTTTGGTGAGAAAGTTTTTTTTAGTG

ATTTAAGAGGTGTGTTTTGGGGTATGGGATTTTTTGGTTT

6#

AGGTGTTTTGTTTTTGAAGAAAAGAGGTGGGTGGATGGGGTGGGGGGGGGGGTGGGAGGCGGGGGTTTGTGGAGTTGCGTTTACGTTTGTATTTGTTGGTTTTTGTTTTGATCGTGAACGTTTAAGGTTGTTGTATGTAGGTTTTTTTTGGTATTATAGGTTTTTTTTTTTTTTTAGGTTTTTTTGAGCGTTTTTGCGGGTTTTTTGGGTTTTATGAGGGGTGGTTGGTGGGTGGGGAGTGTGATTTATTTTTGGTGAGAAAGTTTTTTTTAGTG

ATTTAAGAGGTGTGTTTTGGGGTATGGGATTTTTTGGTTT

**Bisulfite genomic sequence of Jurkat cells**

1#

AGGTGTTTTGTTTTTGAAGAAAAGAGGTGGGTGGATGGGGTGGGGGGGGGGGTGGGAGGTGGGGGTTTGTGGAGTTGCGTTTATGTTTGTATTTGTTGGTTTTTGTTTTGATCGCGAATGTTTAAGGTTGTCGTATGTAGGTTTTTTTTGGTATTATAGGTTTTTTTTTTTTTTTAGGTTTTTTTGAGTGTTTTTGTGGGTTTTTTGGGTTTTATGAGGGGTGGTTGGCGGGTGGGGAGTGTGATTTATTTTTGGTGAGAAAGTTTTTTTTAGTG

ATTTAAGAGGTGTGTTTTGGGGTATGGGATTTTTCGGTTT

2#

AGGTGTTTTGTTTTTGAAGAAAAGAGGTGGGTGGATGGGGTGGGGGGGGGGGTGGGAGGTGGGGGTTTGTGGAGTTGCGTTTATGTTCGTATTTGTTGGTTTTTGTTTTGATTGCGAATGTTTAAGGTTGTCGTATGTAGGTTTTTTTTGGTATTATAGGTTTTTTTTTTTTTTTAGGTTTTTTTGAGTGTTTTTGTGGGTTTTTTGGGTTTTATGAGGGGTGGTTGGCGGGTGGGGAGTGTGATTTATTTTCGGTGAGAAAGTTTTTTTTAGTG

ATTTAAGAGGTGTGTTTTGGGGTATGGGATTTTTTGGTTT

3#

AGGTGTTTTGTTTTTGAAGAAAAGAGGTGGGTGGATGGGGTGGGGGGGGGGGTGGGAGGTGGGGGTTTGTGGAGTTGTGTTTATGTTTGTATTTGTTGGTTTTTGTTTTGATCGCGAATGTTTAAGGTTGTTGTATGTAGGTTTTTTTTGGTATTATAGGTTTTTTTTTTTTTTTAGGTTTTTTTGAGTGTTTTTGCGGGTTTTTTGGGTTTTATGAGGGGTGGTTGGCGGGTGGGGAGTGTGATTTATTTTCGGTGAGAAAGTTTTTTTTAGTG

ATTTAAGAGGTGTGTTTTGGGGTACGGGATTTTTTGGTTT

4#

AGGTGTTTTGTTTTTGAAGAAAAGAGGTGGGTGGATGGGGTGGGGGGGGGGGTGGGAGGCGGGGGTTTGTGGAGTTGCGTTTATGTTTGTATTTGTTGGTTTTTGTTTTGATCGTGAATGTTTAAGGTTGTCGTATGTAGGTTTTTTTTGGTATTATAGGTTTTTTTTTTTTTTTAGGTTTTTTTGAGTGTTTTTGTGGGTTTTTTGGGTTTTATGAGGGGCGGTTGGTGGGTGGGGAGTGTGATTTATTTTTGGTGAGAAAGTTTTTTTTAGTG

ATTTAAGAGGTGTGTTTTGGGGTATGGGATTTTTTGGTTT

5#

AGGTGTTTTGTTTTTGAAGAAAAGAGGCGGGTGGATGGGGTGGGGGGGGGGGTGGGAGGCGGGGGTTTGTGGAGTTGTGTTTATGTTTGTATTTGTTGGTTTTTGTTTTGATCGCGAATGTTTAAGGTTGTTGTATGTAGGTTTTTTTTGGTATTATAGGTTTTTTTTTTTTTTTAGGTTTTTTTGAGTGTTTTTGTGGGTTTTTTGGGTTTTATGAGGGGTGGTTGGCGGGTGGGGAGTGTGATTTATTTTTGGTGAGAAAGTTTTTTTTAGTG

ATTTAAGAGGTGTGTTTTGGGGTATGGGATTTTTCGGTTT

6#

AGGTGTTTTGTTTTTGAAGAAAAGAGGTGGGTGGATGGGGTGGGGGGGGGGGTGGGAGGCGGGGGTTTGTGGAGTTGCGTTTATGTTTGTATTTGTCGGTTTTTGTTTTGATCGTGAATGTTTAAGGTTGTTGTATGTAGGTTTTTTTTGGTATTATAGGTTTTTTTTTTTTTTTAGGTTTTTTTGAGTGTTTTTGCGGGTTTTTTGGGTTTTATGAGGGGTGGTTGGTGGGTGGGGAGTGTGATTTATTTTTGGTGAGAAAGTTTTTTTTAGTG

ATTTAAGAGGTGTGTTTTGGGGTACGGGATTTTTTGGTTT
